# Course of Mental Disorders in Early Cancer Survivorship in Relation to Socioeconomic Status: A Multi‐Center Prospective Longitudinal Study (LUPE)

**DOI:** 10.1002/pon.70059

**Published:** 2025-01-08

**Authors:** Franziska Springer, Ute Goerling, Tanja Zimmermann, Jochen Ernst, Christoph Engel, Myriel Hermann, Peter Esser, Beate Hornemann, Ulrich Keilholz, Florian Lordick, Olaf von dem Knesebeck, David Kissane, Anja Mehnert‐Theuerkauf

**Affiliations:** ^1^ Department of Medical Psychology and Medical Sociology Comprehensive Cancer Center Central Germany (CCCG) University Medical Center Leipzig Leipzig Germany; ^2^ Charité – Universitätsmedizin Berlin Corporate Member of Freie Universität Berlin Humboldt‐Universität zu Berlin Berlin Institute of Health Charité Comprehensive Cancer Center Berlin CharitGermany Germany; ^3^ Department of Psychosomatic Medicine and Psychotherapy Hannover Medical School Hannover Germany; ^4^ Institute for Medical Informatics, Statistics, and Epidemiology (IMISE) Leipzig University Medical Faculty Leipzig Germany; ^5^ Comprehensive Cancer Center University Clinic Centre Dresden Dresden Germany; ^6^ National Center for Tumor Diseases (NCT) Berlin Berlin Germany; ^7^ Department of Medicine II (Oncology, Gastroenterology, Hepatology, and Pulmonology) Comprehensive Cancer Center Central Germany (CCCG) University of Leipzig Medical Center Leipzig Germany; ^8^ Institute of Medical Sociology University Medical Center Hamburg‐Eppendorf Hamburg Germany; ^9^ School of Medicine University of Notre Dame Australia Sydney Australia; ^10^ Departments of Palliative Care Cabrini Health Melbourne Australia; ^11^ School of Clinical Sciences Monash Health and Monash University Melbourne Australia

**Keywords:** cancer, health disparities, mental disorder, oncology, socioeconomic status

## Abstract

**Objective:**

Individuals with low socioeconomic status (SES) exhibit higher rates of mental disorders; however, data in oncological populations are insufficient. This study investigated the course of DSM‐5 mental disorders in cancer patients, stratified by SES, over a period of 1.5 years following initial cancer diagnosis.

**Methods:**

This multi‐center prospective longitudinal study assessed cancer patients within two months of cancer diagnosis (t1), and at 6‐, 12‐, and 18‐month follow‐up (t2–t4) using the SCID‐5 interview for mental disorders based on DSM‐5 criteria. Chi‐square‐tests were tested for frequency changes over time. A generalized linear mixed model (GLMM) was applied with fixed effects for SES and time on mental disorders.

**Results:**

Out of 1030 patients with a SCID‐5 at baseline (53.2% men, 60 years), 821, 719 and 654 participated at respective follow‐ups. The most common diagnoses were skin and prostate cancer. Point prevalence of mental disorders was 20.9% at baseline, decreasing to 18.2%, 14.6%, and 15.0% (t2–t4; *χ* ^2^ (3) = 15.3, *p* = 0.002). Patients with low SES consistently showed highest prevalence rates, whereas patients with high SES showed decreasing rates of mental disorders over time, with a main effect of time (*χ* ^2^ (3) = 19.9, *p* < 0.001) and SES (*χ* ^2^ (2) = 8.8, *p* = 0.01) in the GLMM. Two thirds never met diagnostic criteria for a mental disorder. Sensitivity analysis among study completers (*n* = 592) revealed a similar pattern to the main analysis.

**Conclusions:**

Cancer patients with low SES exhibit impaired coping with cancer‐related stressors, increasing their risk for mental disorders. Social disparities affect physical and mental health, possibly via health behavior or health literacy, and need to be addressed by tailored survivorship care planning.

## 1 Background

Adjustment to cancer‐related stressors varies widely and is influenced by several factors, including psychosocial resources, disease‐related and physical factors, as well as socioeconomic conditions [1]. Mental disorders are therefore common among cancer patients, with approximately 20%–30% meeting the diagnostic criteria for a mental disorder [2, 3, 4]. Variability in the prevalence of mental disorders is often attributed to medical aspects such as cancer type and disease stage, or to psycho‐social factors such as age, income, or social support [5, 6, 7]. Mental disorders are highest shortly after the cancer diagnosis, tend to decrease over time [8, 9], and are associated with increased mortality [10, 11] and higher healthcare costs [12, 13]. However, large epidemiological studies investigating the course of mental disorders following a cancer diagnosis in a longitudinal design are very scarce to date.

The relationship between health and socioeconomic status (SES), measured via education, income and occupation, has been investigated in previous studies, showing worse health outcomes and a higher mental health burden in patients with low SES [14, 15, 16, 17]. However, studies have mainly focused on diseases other than cancer, and data in oncological populations are insufficient to date. Even though patients with low SES are vulnerable for mental health problems and vice versa, they are underrepresented in clinical trials [18].

We have recently reported a four‐week prevalence for DSM‐5 mental disorders of 20.9% in a sample of newly diagnosed cancer patients within two months of cancer diagnosis, most common being depression (9.9%), trauma‐ and stress‐related disorders (6.3%) and anxiety disorders (4.2%) [4]. There were no SES‐related differences in mental disorders during this early survivorship phase, when patients are still within the context of structured clinical care. However, while the initial stress may be equally high for all patients, long‐term coping may vary depending on socioeconomic resources, such as social support, financial resources, and stigma‐free professional support. Longitudinal studies investigating the course of mental disorders following a cancer diagnosis are scarce, and the impact of the SES on coping with cancer‐related stressors is largely unknown. This, however, would be highly valuable for better psycho‐oncological care planning.

The aims of the present study therefore were (1) to provide point prevalence rates of DSM‐5 mental disorders within a large sample of newly diagnosed cancer patients over a period of 1.5 years following initial cancer diagnosis, (2) to stratify the prevalence rates according to SES, and (3) to describe the course of mental disorders over time based on baseline status of mental disorder.

## 2 Methods

### 2.1 Study Design and Participants

A prospective multi‐center longitudinal observational study was conducted among patients newly diagnosed with cancer. Participants were assessed at four measurement points, that is at baseline within two months of initial cancer diagnosis (t1), at 6‐month (t2), 12‐month (t3), and 18‐month follow‐up (t4). Patients were eligible if they had been diagnosed with a malignant solid tumor according to their medical records (ICD‐10: C00‐C80), were ≥ 18 years and scheduled for cancer treatment at the study centers, had sufficient German language skills and were physically, psychologically and cognitively able to participate. Patients were excluded if they had been diagnosed with a second or relapsed cancer diagnosis. Written informed consent was provided by all participants prior to study participation.

The study was conducted at three German university cancer centers (Leipzig, Berlin, Hannover) and cooperating cancer centers (Braunschweig, Dresden, Göttingen). Ethics approval was obtained (207/18‐ek, SR‐EK‐536112020, 8533_BO_K_2019, 14/4/21Ü) and the study was registered in the International Clinical Trials Registry (NCT04620564). The study protocol has been published [19].

### 2.2 Procedures

Patients were recruited between April 2020 and July 2022 and data collection was completed by January 2024. Patients were systematically assessed for eligibility via screening of their medical records by the research team. Eligible patients were approached by a study member while receiving inpatient care. Patients were provided with written information about the study. If patients could not be contacted directly during inpatient care due to COVID‐19 restrictions, they received an invitation letter along with study materials. Patients who declined study participation were asked to provide sociodemographic and medical information, as well as reasons for their non‐participation.

Participants were assessed at all measurement points (t1–t4) with a Structured Clinical Interview for DSM‐5 mental disorders (SCID‐5, clinical version) [20], that was administered face‐to‐face or over the phone, and received validated paper‐pencil or online questionnaires, to be filled in at the cancer center or remotely at home. Reminders were given via phone after two and three weeks if the questionnaire had not been returned.

### 2.3 Measures

SCID‐5 interviews were conducted to assess point prevalence rates of current mental disorders at each measurement point based on DSM‐5 criteria. The following diagnoses were covered in the interview: (i) mood episodes and persistent depressive disorder, (ii) substance use disorders, (iii) anxiety disorders, (iv) obsessive‐compulsive and related disorders and posttraumatic stress disorder, and (v) adjustment disorder. Study interviewers underwent standardized training. The quality of interviewers was ensured by several test interviews, with one being videotaped and evaluated by a certified psychotherapist at the University Medical Center Leipzig.

Medical data were extracted from medical records, in addition to self‐reported answers in the questionnaires. Sociodemographic data were collected during the SCID‐5 interview. Based on information regarding education, income and occupation, the Winkler Stolzenberg index [21] for SES was calculated at baseline and was grouped into low, medium and high SES. Physical functionality was assessed with the Karnofsky performance status.

### 2.4 Statistical Analyses

Baseline characteristics are displayed descriptively. Non‐responder analyses and drop‐out analyses were performed on relevant sociodemographic and medical factors using Chi‐square‐ and *t*‐tests. Non‐responder analyses were performed for patients excluded because of insufficient data to check full eligibility criteria (*n* = 1575) and for those who refused to participate (*n* = 552).

Point prevalence rates for mental disorders in the total sample were SES‐weighted according to the sample's SES distribution to correct for over‐/under‐sampling relative to the distribution in the German adult population [22] (20% low, 60% medium, 20% high SES). Each patient was assigned a design weight and survey‐weighted means for the prevalence rates with 95% confidence intervals were calculated using the “survey” R package. Point prevalence rates for mental disorders in the SES subgroups were reported unweighted as raw values.

Chi‐square‐tests assessed changes in frequencies over time within each SES group. A Generalized Linear Mixed Model (GLMM) with a binomial distribution was fitted to examine the effects of SES and time on the binary outcome mental disorder, and random intercepts for subjects to account for repeated measures. Interaction between time and SES were tested and reported if significant. All analyses were conducted for any mental disorder and different types of mental disorders.

A sensitivity analysis was performed considering only study completers (*n* = 592) with SCID‐5 data at all measurement points. In addition, descriptive analyses for the course of mental disorders are displayed within this subgroup, i.e. patients with persistent mental disorders, improvements in mental disorders, incident mental disorders, and patients without a mental disorder at any time.

All analyses were performed with R statistics software, version 4.3.1 [23]. Statistical tests were two‐tailed with *α* = 5%.

## 3 Results

### 3.1 Participants

**FIGURE 1:**
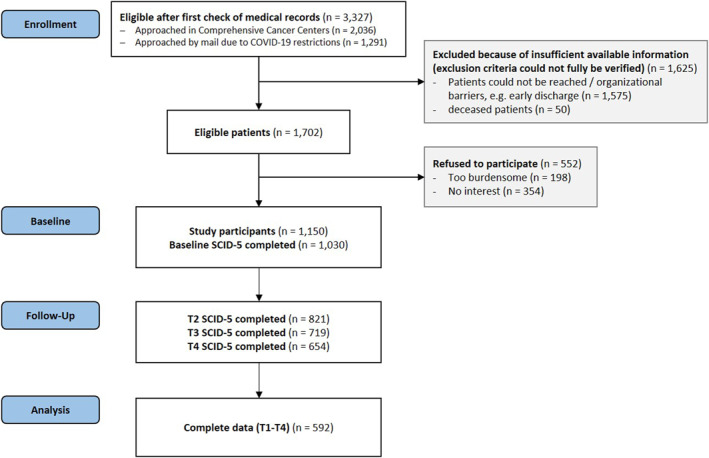
Flow‐diagram.

After the initial eligibility check using medical record information, 3327 patients were found to be eligible. A thorough eligibility assessment was then conducted by contacting the patients during their inpatient stay or via phone. In total, 1575 patients could not be reached to verify final eligibility (e.g. language ability). Of the remaining 1702 eligible patients, 1150 (67.6%) were included in the study. In total, 1030 patients completed the SCID‐5 interview at baseline and were considered for further analyses. SCID‐5 data at follow‐ups were provided by 821, 719 and 654 participants, respectively (Figure 1). Complete SCID‐5 data at all measurement points were available for 592 participants.

Compared to study participants (*n* = 1150), non‐responders who were excluded due to insufficient data to assess eligibility (*n* = 1575) were older (60.4 vs. 63.4 years, *p* < 0.001) and more likely to be male (53.9% vs. 58.0%, *p* = 0.04). Data on SES and physical functionality were not available for this group. Non‐responders who refused to participate (*n* = 552) did not differ in gender (*p* = 0.49) but were more likely to have a lower SES (low: 16.6% vs. 27.4%; high: 38.9% vs. 26.8%, *p <* 0.001), were older (60.4 vs. 65.9 years, *p* < 0.001), and had worse physical functionality (80.0 vs. 73.6, *p <* 0.001), compared to study participants.

Study completers (*n* = 592) and non‐completers (*n* = 438) did not differ in age (*p* = 0.97), gender (*p* = 0.09), partnership (*p* = 0.62), distress (*p* = 0.40) and mental disorder at baseline (*p* = 0.10). However, study completers had a higher SES (low: 14.5% vs. 19.6%; high: 43.2% vs. 35.2%, *p* = 0.01) and a less progressed disease (UICC IV: 8.7% vs. 20.1%, *p <* 0.001).

### 3.2 Sample Characteristics

Participants were on average 60 years old, 53.2% were male. The most frequent diagnoses were skin (18.8%) and prostate cancer (17.2%) (Table 1). In total, 16.7% of the participants were classified as having low SES, 43.5% as medium SES, and 39.8% as high SES. Sociodemographic differences were found in SES groups, with men having a higher SES (low: 14.6% vs. 19.1%; high: 44.5% vs. 34.4%, *p* = 0.003) and participants with a partner having a higher SES (low: 12.2% vs. 33.7%; high: 44.3% vs. 22.8%, *p* < 0.001). There were no differences in self‐reported utilization of psychological/psychotherapeutic support between SES groups at any measurement point.

**TABLE 1:** Baseline sample characteristics (*n* = 1030).

	%	(*n*)
Age, years mean (SD, range)	60.2 (13.3, 19–92)	
Gender		
Men	53.2	(548)
Women	46.8	(482)
Marital status		
Married	64.8	(662)
Single	17.1	(175)
Divorced/Separated	11.9	(121)
Widowed	6.2	(63)
Partnership	80.1	(813)
Educational qualification		
University degree	42.1	(432)
High school/Vocational training	54.5	(560)
No qualification	3.4	(35)
Occupation		
Employed	49.9	(508)
Retired	44.8	(457)
Unemployed	2.6	(27)
Other	2.6	(27)
Socioeconomic status		
Low	16.7	(172)
Medium	43.5	(448)
High	39.8	(410)
Residential area		
Urban (≥ 20.000 inhabitants)	56.8	(583)
Rural (< 20.000 inhabitants)	43.2	(444)
Tumor site		
Skin (C43‐C44)	18.8	(194)
Prostate (C61)	17.2	(177)
Digestive organs (C15‐C26)	15.3	(158)
Female genital organs (C51‐C58)	13.1	(135)
Breast (C50)	12.5	(129)
Kidney/urinary tract (C64‐C68)	7.3	(75)
Head and neck (C00‐C14)	5.2	(54)
Lung (C34)	3.0	(31)
Other	7.5	(77)
Months since diagnosis^a^, Mean, Median (range)	1.4, 1.0 (0–6)	
≤ 2 months	86.8	(894)
> 2 months	13.2	(136)
UICC Stage		
I	42.8	(441)
II	21.5	(221)
III	16.8	(173)
IV	12.7	(131)
Not determinable^b^	6.2	(64)
Cancer treatment^c^		
Surgery	87.3	(876)
Radiotherapy	16.0	(161)
Chemotherapy	20.6	(207)
Other	12.0	(120)
Physical functionality (Karnofsky), Mean (SD, range)	80.45 (21.3, 10–100)	

*Note:* (*n*) are valid answers only, with deviations from the full sample size being missing values; percentages are based on valid answers.

Abbreviations: SD, standard deviation; UICC, Union for International Cancer Control disease stage.

^a^ months since diagnosis in relation to first questionnaire completion. > 2 months: deviation from the study protocol (maximum up to 6 months), since recruitment during COVID‐19 was only possible indirectly via mail, which extended the time required for the study inclusion process.

^b^ not determinable, for example basalioma.

^c^ multiple responses possible; based only on patients who received a cancer treatment (*n* = 1004).

### 3.3 Point Prevalence of Mental Disorders Stratified by SES

**FIGURE 2:**
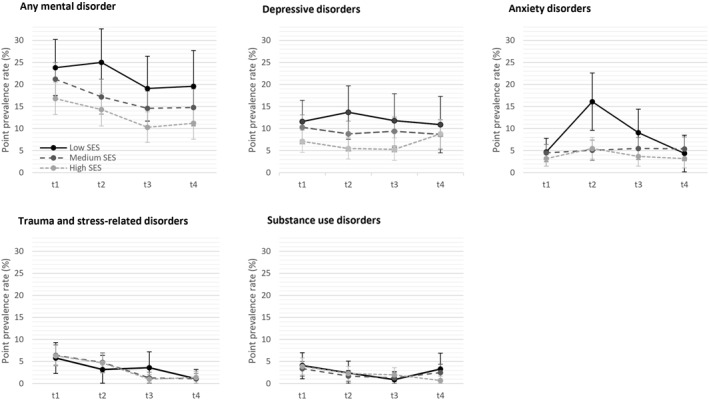
Point prevalence rates for mental disorders over time (t1: Baseline within 2 months after cancer diagnosis, t2: 6 months, t3: 12 months, t4: 18 months), stratified by socioeconomic status (SES) with 95% confidence intervals.

The SES‐weighted prevalence of any mental disorder in the total sample was 20.9% at baseline (t1), significantly decreasing (*p* = 0.002) to 18.2% at 6‐month (t2), 14.6% at 12‐month (t3), and 15.0% at 18‐month follow‐up (t4) (Table 2). Patients with low SES had the highest prevalence rates at each measurement point (Figure 2), with 23.8% meeting the criteria for a mental disorder at baseline (t1), and 19.6% at t4 (Table 3, *p* = 0.61). Patients with high SES had the lowest prevalence rates, starting with 16.8% at baseline and decreasing to 11.2% at t4 (*p* = 0.048). The Wald chi‐square test of the GLMM indicated a main effect for time (*p* < 0.001) and SES (*p* = 0.01) on mental disorder (Table 3).

**TABLE 2:** Point prevalence rates for mental disorders over time, stratified by socioeconomic status (SES).

	t1 (baseline)		t2 (6 months)		t3 (12 months)		t4 (18 months)	
	*n*	% [CI 95%]	*n*	% [CI 95%]	*n*	% [CI 95%]	*n*	% [CI 95%]
**Any mental disorder**								
All participants^a^	1030	20.9 [18.1–23.6]	821	18.2 [15.3–21.1]	719	14.6 [11.8–17.5]	654	15.0 [11.9–18.1]
Low SES	172	23.8 [17.5–30.2]	124	25.0 [17.4–32.6]	110	19.1 [11.7–26.4]	92	19.6 [11.5–27.7]
Medium SES	448	21.2 [17.4–25.0]	354	17.2 [13.3–21.2]	309	14.6 [10.6–18.5]	277	14.8 [10.6–19.0]
High SES	410	16.8 [13.2–20.5]	343	14.3 [10.6–18.0]	300	10.3 [6.9–13.8]	285	11.2 [7.6–14.9]
**Depressive disorders**								
All participants	1030	9.9 [7.9–11.9]	821	9.1 [6.9–11.3]	719	9.1 [6.7–11.4]	654	9.1 [6.7–11.5]
Low SES	172	11.6 [6.8–16.4]	124	13.7 [7.6–19.7]	110	11.8 [5.8–17.9]	92	10.9 [4.5–17.3]
Medium SES	448	10.3 [7.5–13.1]	354	8.8 [5.8–11.7]	309	9.4 [6.1–12.6]	277	8.7 [5.3–12.0]
High SES	410	7.1 [4.6–9.6]	343	5.5 [3.1–8.0]	300	5.3 [2.8–7.9]	285	8.8 [5.5–12.1]
**Anxiety disorders**								
All participants	1030	4.2 [2.9–5.6]	821	7.4 [5.4–9.3]	719	5.9 [3.9–7.8]	654	4.7 [2.9–6.6]
Low SES	172	4.7 [1.5–7.8]	124	16.1 [9.6–22.6]	110	9.1 [3.7–14.4]	92	4.4 [0.2–8.5]
Medium SES	448	4.5 [2.5–6.4]	354	5.1 [2.8–7.4]	309	5.5 [3.0–8.0]	277	5.4 [2.7–8.1]
High SES	410	3.2 [1.5–4.9]	343	5.5 [3.1–8.0]	300	3.7 [1.5–5.8]	285	3.2 [1.1–5.2]
**Trauma and stress‐related disorders**								
All participants	1030	6.3 [4.7–7.9]	821	4.5 [2.9–6.0]	719	1.7 [0.1–2.8]	654	1.2 [0.2–2.0]
Low SES	172	5.8 [2.3–9.3]	124	3.2 [0.1–6.3]	110	3.6 [0.1–7.2]	92	1.1 [0.0–3.2]
Medium SES	448	6.4 [4.2–8.8]	354	4.8 [2.6–7.0]	309	1.3 [0.0–2.6]	277	1.1 [0.0–2.3]
High SES	410	6.3 [4.0–8.7]	343	4.7 [2.4–6.9]	300	1.0 [0.0–2.1]	285	1.4 [0.0–2.8]
**Substance use disorders**								
All participants	1030	3.6 [2.4–4.8]	821	2.0 [0.9–3.0]	719	1.4 [0.5–2.3]	654	2.3 [1.0–3.6]
Low SES	172	4.1 [1.1–7.0]	124	2.4 [0.0–5.1]	110	0.9 [0.0–2.7]	92	3.3 [0.0–6.9]
Medium SES	448	3.4 [1.7–5.0]	354	1.7 [0.4–3.0]	309	1.3 [0.0–2.6]	277	2.5 [0.7–4.4]
High SES	410	3.9 [2.0–5.8]	343	2.3 [0.7–3.9]	300	2.0 [0.4–3.6]	285	0.7 [0.0–1.7]

*Note:* Socioeconomic status (SES), confidence interval (CI).

^a^ Prevalence rates for the total sample were weighted by SES to compensate for over‐ and under‐sampling of SES groups compared to the general population.

**TABLE 3:** Changes in mental disorders over time.

	Within group comparison		Generalized linear mixed model (GLMM)		
	χ^2^ (df)	*p*		χ^2^ (df)	*p*
**Any mental disorder**					
All participants	15.3 (3)	**0.002**	Time	19.9 (3)	**< 0.001**
Low SES	1.8 (3)	0.61	SES	8.8 (2)	**0.01**
Medium SES	7.5 (3)	0.06			
High SES	7.9 (3)	**0.048**			
**Depressive disorders**					
All participants	1.1 (3)	0.78	Time	2.0 (3)	0.57
Low SES	4.8 (3)	0.92	SES	3.0 (2)	0.22
Medium SES	0.7 (3)	0.86			
High SES	3.7 (3)	0.30			
**Anxiety disorders**					
All participants	9.7 (3)	**0.02**	Time	21.2 (3)	**< 0.001**
Low SES	14.7 (3)	**0.002**	SES	4.2 (2)	0.12
Medium SES	0.5 (3)	0.91	Time x SES	17.9 (6)	**0.006**
High SES	3.5 (3)	0.32			
**Trauma and stress‐related disorders**					
All participants	41.6 (3)	**< 0.001**	Time	46.1 (3)	**< 0.001**
Low SES	3.9 (3)	0.28	SES	0.01 (2)	0.99
Medium SES	20.5 (3)	**< 0.001**			
High SES	19.5 (3)	**< 0.001**			
**Substance use disorders**					
All participants	10.0 (3)	**0.02**	Time	20.8 (3)	**< 0.001**
Low SES	2.6 (3)	0.46	SES	0.1 (2)	0.94
Medium SES	4.2 (3)	0.24			
High SES	7.7 (3)	0.053			

*Note:* Generalized Linear Mixed Model (GLMM). Within group comparisons of SES groups over time; GLMM modeling fixed effects of SES and time, and random effects of subjects; significant values marked in bold.

In analyses on different mental disorders the GLMM for *depressive disorders* indicated no effect of time (*p* = 0.57) and SES (*p* = 0.22). For *anxiety disorders* there was a main effect of time (*p* < 0.001), but not SES (*p* = 0.12), as well as an interaction of time and SES (*p* = 0.006). For *trauma and stress‐related disorders* there was a main effect of time (*p* < 0.001), but not SES (*p* = 0.99), as well as for *substance use disorders* (time: *p* < 0.001; SES: *p* = 0.94).

### 3.4 Sensitivity Analyses Among Study Completers

Sensitivity analyses among study completers (*n* = 592) revealed a comparable pattern of results as the main analysis, namely decreasing prevalence rates over time (19.6%, 16.9%, 14.3%, 15.4% at t1–t4), with highest and stable prevalence rates in patients with low SES (Table S1). Within group comparisons did not reach significance. Comparable to the main analysis, there was a main effect for time (χ^2^ (3) = 11.0, *p* = 0.01) but not SES (χ^2^ (2) = 5.9, *p* = 0.052) on mental disorder in the GLMM.

### 3.5 Course of Mental Disorders

In total, 68.1% (*n* = 403) of the patients never met the criteria for a mental disorder at any measurement point (analysis among study completers). Among patients with a mental disorder at baseline (*n* = 107), *persistent mental disorder* was evident in 44.9% (*n* = 48) up until t2, in 29.0% (*n* = 31) up until t3, and in 17.8% (*n* = 19) up until t4. Conversely, more than half of the patients with a mental disorder at baseline showed *improvements in mental disorder* by the 6‐month follow‐up (t2) (55.1%, *n* = 59), with an additional 15.9% (*n* = 17) improving by t3, and another 11.2% (*n* = 12) by t4. Among patients without a mental disorder at baseline (*n* = 485), 9.5% (*n* = 46) *newly developed a mental disorder* by t2, additional 3.3% (*n* = 16) by t3, and further 4.1% (*n* = 20) by t4.

## 4 Discussion

This multi‐center longitudinal observational study investigated the prevalence of mental disorders in cancer patients over 1.5 years after initial cancer diagnosis in relation to SES. Prevalence of mental disorders was highest shortly after the cancer diagnosis and decreased over time, particularly among those with high SES. Prevalence of mental disorders in patients with low SES was continuously the highest and stable over time. Adaptation to cancer‐related stressors varied among different types of mental disorder, with only minor changes in depressive disorders, an increase in anxiety disorders around the end of cancer treatment for patients with low SES, and a decrease over time in trauma and stress‐related disorders and substance use disorders.

The critical life event of a cancer diagnosis is burdensome for many patients; however, approximately two thirds of the patients in our study (68.1%) never met diagnostic criteria for a mental disorder at any time. This aligns well with previous studies [2, 3], showing a prevalence of mental disorders in cancer patients across different healthcare settings at around 30%. The prevailing decrease of mental disorders over time may represent an adaptation to challenges that arise within the first months, such as dealing with anxiety or uncertainty during the treatment, changes in social roles, work and values. Our findings on the overall decreasing temporal pattern of mental disorders are consistent with previous research [8, 9, 24, 25], which has primarily focused on specific cancer entities. A population‐based registry study among different cancer types [8] assessed mental disorders via registered ICD‐10 diagnoses and reported an increase in mental disorders starting a few months before cancer diagnosis, with the peak during the week after the diagnosis, and a decrease thereafter. Comparable to our results, they reported the highest cumulative incidence for depression, anxiety and stress‐related disorders. Complementing their results on elevated risks, our study provides point prevalence rates of mental disorders for a mixed cancer sample in the first 1.5 years of cancer survivorship.

Our study is one of the first to demonstrate that adaptation to cancer‐related stressors is related to socioeconomic resources. While there was no SES‐related difference in mental disorders shortly after the cancer diagnosis [4], improvements in mental disorders over time were evident in patients with higher SES. Rates at diagnosis might be explained by the initial shock, affecting patients independently of their SES, and supportive care within structured clinical care settings. While patients with medium and high SES thereafter improve, possibly due to better access to support services, financial and social resources, effective coping strategies, and better health literacy [26] to manage mental health problems, patients with low SES exhibit impaired adaptation and continued high burden. The end of structured care might thus expose socioeconomic disparities in coping with stressors. Future studies should report on the patients' SES and take extra effort when planning patient recruitment for clinical studies to avoid under‐sampling of low SES, which is often the case [18]. Improving the recruitment of patients with low SES may include several strategies, such as using simple, accessible language for study material and highlighting the value of the patients' expertise to foster a sense of empowerment. Additionally, offering financial incentives and minimizing the time commitment required by ensuring that participation is quick and easy to implement can further encourage participation among this population.

The trajectory of mental disorders displays different patterns depending on the type of disorder. In our study, depressive disorders, affecting around 9% of patients, exhibit no major changes over time. This is supported by previous research indicating that about 10% experience depressive symptoms that remain within the clinical range over time [24, 27, 28]. This may be explained by the nature of depressive symptoms, involving feelings of hopelessness and a negative outlook on the future, as well as a normalization of these symptoms. Living with long‐term consequences of cancer may not induce ongoing anxiety about the disease but may rather affect patients' mood due to the psychological and physical toll of the disease. Patients with higher SES also reported relatively stable rates for anxiety disorders, whereas those with low SES exhibited a vulnerable phase at the 6‐month follow‐up. This period approximately coincides with the end of primary cancer treatment, the transition to managing the disease in daily life and fear of recurrence [29]. The end of primary care may heighten the perceived threat of cancer, particularly among patients with low SES, due to feelings of loss of control, occupational and financial deficits, and poorer access to psychotherapy [30]. Our study is the first to report SES‐weighted prevalence rates of mental disorders and stratifying them by SES, complementing research on anxiety trajectories in cancer [28, 31], which also indicated a high trajectory of anxiety in patients with financial difficulties. In contrast to depressive and anxiety disorders, trauma and stress‐related disorders decreased over time in our study, suggesting that patients, regardless of socioeconomic background, adapt well to initially traumatic or severely stressful events.

### 4.1 Clinical Implications

We have shown, that social disparities affect mental health, that need to be addressed by tailored survivorship care planning for patients with low SES. Particularly supporting these patients with symptoms of anxiety around the end of cancer treatment seems crucial to improve during clinical cancer care. The impact of untreated mental disorders on healthcare outcomes has been thoroughly described, including worse survival rates, reduced healthcare utilization, and increased healthcare costs [10, 13]. Despite the high‐quality, multidisciplinary, structured care offered at German comprehensive cancer centers [32], such as those in our study, which provide free access to psycho‐oncological and social support, social disparities in psychological burden remain evident. One explanation might be that access to supportive care is not adequately suited for patients with low SES. Therefore, mere information about existing support services may not be sufficient when health literacy is low. A targeted survivorship care structure, tailored referrals to psychological support, and standardized clinical pathways for this vulnerable subgroup of patients could potentially improve mental health.

### 4.2 Strengths and Limitations

A major strength of our study is the longitudinal design, which captures early survivorship starting within two months of cancer diagnosis and follows patients for up to 1.5 years after initial cancer diagnosis, with a comparatively high retention rate. In addition, we assessed mental disorders using clinical diagnostic interviews and stratified prevalence rates by SES. Given the results of our non‐responder and drop‐out analyses, and variation from cancer prevalence rates for certain cancers (e.g. lung, breast and skin cancer) [33], our sample may show a bias towards younger, physically functioning patients with higher SES. However, our prevalence rates were weighted according to the SES distribution to compensate for over‐/under‐sampling. Additionally, analyses of study completers supported our main findings. Nevertheless, results should be interpreted with caution, and further studies are needed to validate our findings. A further limitation of our study is that, due to COVID‐19 restrictions and recruitment difficulties, our sample is underpowered, particularly among those with low SES. Findings related to sub‐analyses with smaller sample sizes and large CIs should be interpreted with caution. Longitudinal population‐based studies using clinical diagnostic interviews are needed.

## 5 Conclusion

Cancer patients with low socioeconomic resources are at higher risk for mental disorders during the first 1.5 years of cancer survivorship and exhibit impaired adaptation to cancer‐related stressors. Social disparities impact physical and mental health, potentially through health behavior or health literacy. Our findings highlight the need for enhanced monitoring, adapted referral processes within survivorship care, and tailored support services for this vulnerable patient group.

## Author Contributions

Conceptualization: A.M.‐T., J.E., U.G., T.Z. Funding acquisition: A.M.‐T., J.E., U.G., T.Z., P.E. Resources: F.L., U.K. Data curation: F.S., J.E. Formal analysis: F.S., C.E. Supervision: A.M.‐T., D.K., Ov.dK. Visualization: F.S. Writing–original draft: F.S. Writing–review & editing: all authors.

## Conflicts of Interest

The authors declare no conflicts of interest.

## Declaration of Generative AI in Scientific Writing

During the preparation of this work the author(s) used DeepL for quality checking of language. After using this tool/service, the author(s) reviewed and edited the content as needed and take(s) full responsibility for the content of the publication.

## Supporting information

Table S1

## Data Availability

The data that support the findings of this study are available from the corresponding author upon reasonable request. All of the individual participant data collected during the trial and after de‐identification, and the study protocol will be shared immediately following publication and ending 36 months following article publication. Data will be shared with researchers who provide a methodologically sound proposal, to achieve the aims in the approved proposal. Proposal should be directed to the last author (anja.mehnert@medizin.uni-leipzig.de); to gain access, data requestors will need to sign a data access agreement.
