# Supplementary material for: Course of Mental Disorders in Early Cancer Survivorship in Relation to Socioeconomic Status: A Multi‐Center Prospective Longitudinal Study (LUPE)
Source: Psychooncology. 2025 Jan 8;34(1):e70059. doi: 10.1002/pon.70059 (PMC11711303; doi:10.1002/pon.70059)
Supplement: Supplementary file 1 — Table S1 [file PON-34-e70059-s001.docx]

**SUPPLEMENTARY MATERIAL**

| **Table S1. Study completers (n=592) prevalence rates for any mental disorder over time, stratified by socioeconomic status (SES)** | | | | | | | |
| --- | --- | --- | --- | --- | --- | --- | --- |
|  | **t1**  **(Baseline)** | | **t2**  **(6 months)** | **t3**  **(12 months)** | **t4**  **(18 months)** | **Within group comparison** | |
|  | n | % (n) [CI 95%] | % [CI 95%] | % [CI 95%] | %  [CI 95%] | Ꭓ^2^ (df) | *p* |
| **All participants**^†^ | 592 | 19.6  [16.0-23.1] | 16.9 [13.5-20.2] | 14.3 [11.1-17.5] | 15.4 [12.2-18.7] | 6.7 (3) | 0.08 |
| **Low SES** | 86 | 19.8  [11.4-28.2] | 20.9 [12.3-29.5] | 19.8 [11.4-28.2] | 19.8 [11.4-28.2] | 0.1 (3) | 0.997 |
| **Medium SES** | 250 | 21.2  [16.1-26.3] | 16.8 [12.2-21.4] | 14.0 [9.7-18.3] | 15.2 [10.7-19.7] | 5.3 (3) | 0.15 |
| **High SES** | 256 | 14.5  [10.1-18.8] | 13.3 [9.1-17.4] | 9.8 [6.1-13.4] | 11.7 [7.8-15.7] | 2.9 (3) | 0.40 |
| Socioeconomic status (SES), confidence interval (CI);  † Prevalence rates for the total sample were weighted by SES to compensate for over- and under-sampling of SES groups compared to the general population | | | | | | | |
